# Inhibition of Endothelin-1-Mediated Contraction of Hepatic Stellate Cells by FXR Ligand

**DOI:** 10.1371/journal.pone.0013955

**Published:** 2010-11-11

**Authors:** Jiang Li, Ramalinga Kuruba, Annette Wilson, Xiang Gao, Yifei Zhang, Song Li

**Affiliations:** Department of Pharmaceutical Sciences, School of Pharmacy, Center for Pharmacogenetics, University of Pittsburgh, Pittsburgh, Pennsylvania, United States of America; University of Western Ontario, Canada

## Abstract

Activation of hepatic stellate cells (HSCs) plays an important role in the development of cirrhosis through the increased production of collagen and the enhanced contractile response to vasoactive mediators such as endothelin-1 (ET-1). The farnesoid X receptor (FXR) is a member of the nuclear receptor superfamily that is highly expressed in liver, kidneys, adrenals, and intestine. FXR is also expressed in HSCs and activation of FXR in HSCs is associated with significant decreases in collagen production. However, little is known about the roles of FXR in the regulation of contraction of HSCs. We report in this study that treatment of quiescent HSCs with GW4064, a synthetic FXR agonist, significantly inhibited the HSC transdifferentiation, which was associated with an inhibition of the upregulation of ET-1 expression. These GW4064-treated cells also showed reduced contractile response to ET-1 in comparison to HSCs without GW4064 treatment. We have further shown that GW4064 treatment inhibited the ET-1-mediated contraction in fully activated HSCs. To elucidate the potential mechanism we showed that GW4064 inhibited ET-1-mediated activation of Rho/ROCK pathway in activated HSCs. Our studies unveiled a new mechanism that might contribute to the anti-cirrhotic effects of FXR ligands.

## Introduction

Hepatic fibrosis or cirrhosis is a chronic scarring process of the liver that is associated with increased and altered deposition of extracellular matrix (ECM). During the chronic liver injury, hepatic stellate cells (HSCs) undergo a process of transdifferentiation from a resting, fat-storing phenotype to a myofibroblast-like phenotype characterized by expression of fibroblastic cell markers such as α-smooth muscle actin (α-SMA). Activated HSCs produce increased amounts of ECM components that contribute significantly to the fibrotic changes in cirrhosis [1], [2]. In addition, activated HSCs gain contractile phenotype and increasing evidence suggests that the contractile force generated by HSCs contributes to the regulation of sinusoidal blood flow and the development of portal hypertension [3]. A number of vasoactive molecules can trigger contractile response in HSCs with endothelin-1 being the most potent constrictor [4], [5], [6]. The regulation of HSC activation during the chronic liver injury is not completely understood, however, a number of studies have suggested that members of the nuclear receptor (NR) superfamily negatively regulate HSC transdifferentiation [7], [8], [9].

The farnesoid X receptor (FXR) belongs to the superfamily of ligand-activated transcriptional factors. FXR has the typical nuclear receptor structure and can be activated by structurally different ligands, including several primary and secondary bile acid (BA) species conjugated to either taurine or glycine [10]. In addition to BAs, a number of synthetic FXR agonists have been developed, among which GW4064 is one of the most potent FXR agonists [11], [12]. FXR is highly expressed in liver, kidney, adrenals, and intestine and plays a key role in the homeostasis of cholesterol and BAs via regulation of the expression of the genes that are involved in the synthesis and transport of BAs [13], [14], [15]. FXR is also expressed in HSCs and activation of FXR has been shown to negatively regulate HSC transdifferentiation, which is associated with a significant inhibition of both basal and TGF-β-induced collagen production [16]. Treatment with a FXR ligand significantly inhibited the activation of HSCs and a concomitant decrease in collagen deposition in a rat model of CCl_4_-induced liver injury [17]. However, little is known about the roles of FXR in the regulation of contraction of HSCs. We report in this study that treatment of quiescent HSCs with GW4064, a synthetic FXR agonist, significantly inhibited the HSC transdifferentiation, which was associated with an inhibition of the upregulation of ET-1 expression. These GW4064-treated cells also showed reduced contractile response to ET-1 in comparison to untreated HSCs. We have further shown that GW4064 treatment inhibited the ET-1-mediated contraction in fully activated HSCs. Our studies unveiled a new mechanism that might contribute to the anti-cirrhotic effects of FXR ligands.

## Results

### GW4064 treatment inhibited transdifferentiation of HSCs

**Figure 1:**
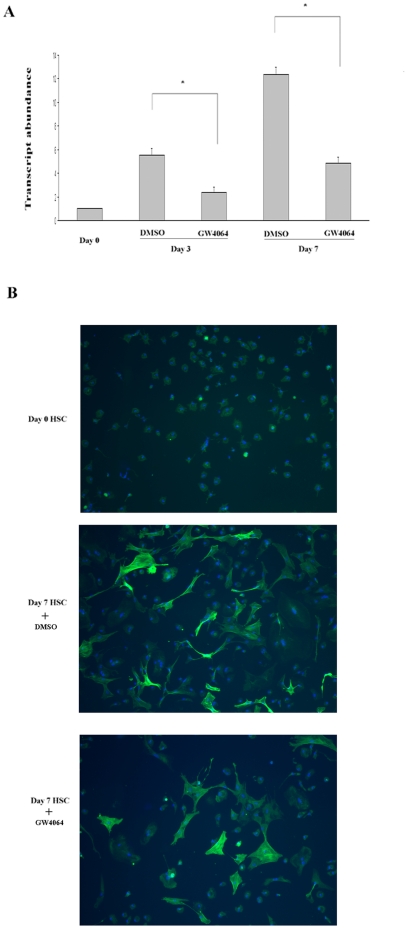
GW4064 treatment inhibited transdifferentiation of HSCs. Freshly isolated rat HSCs were cultured for 7 days in 10% FCS alone (control) or 10% FCS medium containing 1 µmol/L GW4064. The expression of α-SMA mRNA (A) and protein (B) was examined by real-time RT-PCR and immunofluorescence staining, respectively. Data are mean ±SE of 6 experiments. *P<.001 vs. control cells.

Freshly isolated rat HSCs transdifferentiated to a myofibroblastic phenotype *in vitro* when cultured on plastic in serum-containing media, as evidenced by an induction of α-SMA at mRNA (Fig. 1A) and protein level (Fig. 1B). This activation process was significantly inhibited when the cells were continuously treated with GW4064. Our results were in agreement with those by Fiorucci and colleagues with different FXR ligands [17].

### GW4064 treatment inhibited the upregulation of ET-1 expression during HSC activation

**Figure 2:**
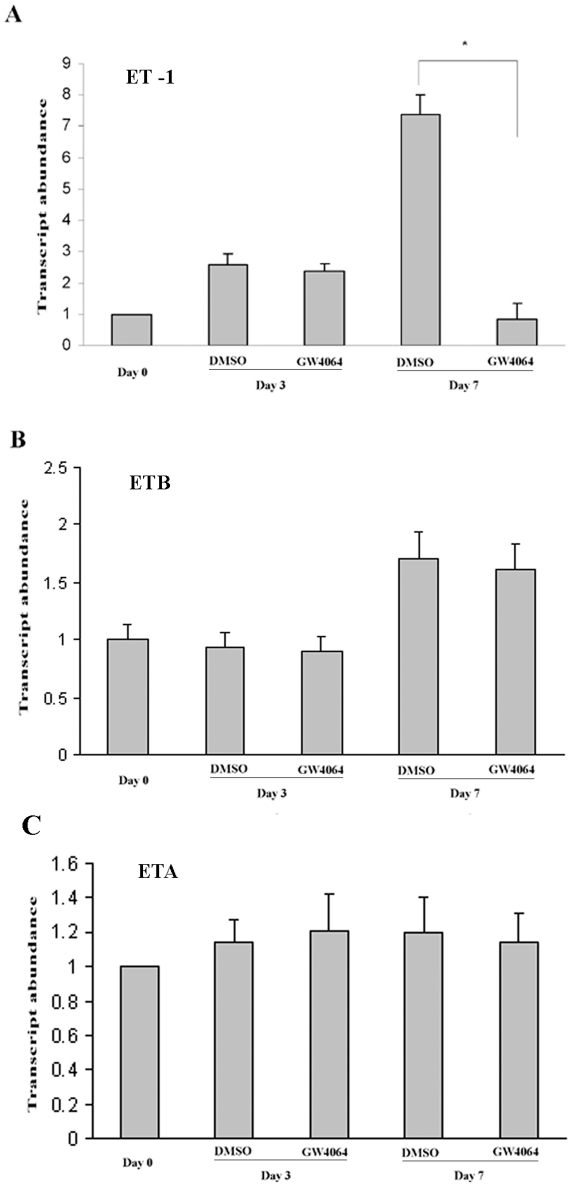
GW4064 treatment inhibited the upregulation of ET-1 expression during HSC activation. Freshly isolated rat HSCs were cultured for 7 days in 10% FCS alone (control) or 10% FCS medium containing 1 µmol/L GW4064. The mRNA expression levels of ET-1 (A), ETB (B) and ETA (C) were examined by real-time RT-PCR, respectively. Data are mean ±SE of 6 experiments. *P<.001 vs. control cells.

It has been shown that ET-1 expression is significantly upregulated during HSC transdifferentiation. A similar result was shown in our study with rat HSCs as examined by real-time RT-PCR (Fig. 2A). This upregulation was significantly inhibited when HSCs were continuously treated with GW4064.

Fig. 2B also shows that transdifferentiation of HSCs was associated with upregulation of ETB receptor. This is in agreement with the study by Chi and colleagues [18]. However, GW4064 treatment had no effects on changes of ETB expression during the activation of HSCs. GW4064 had no effect either on the expression levels of ETA during the transdifferentiation (Fig. 2C).

### GW6064 treatment of HSCs led to reduced contractile response to ET-1

**Figure 3:**
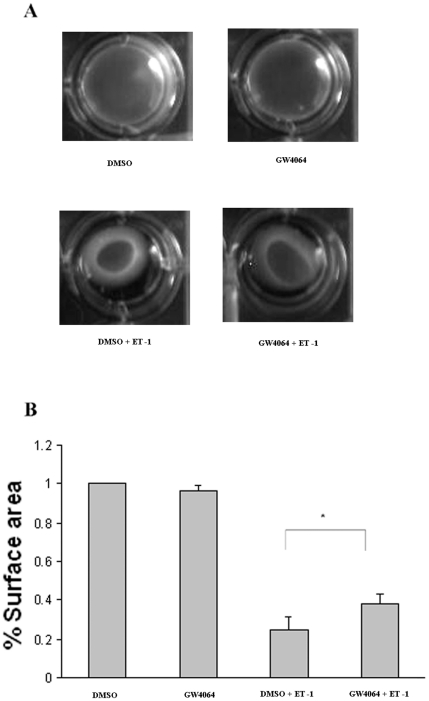
GW4064 treatment of HSCs led to reduced contractile response to ET-1. Freshly isolated rat HSCs were cultured for 7 days in 10% FCS medium alone (control) or 10% FCS medium containing 1 µmol/L GW4064. Collagen gel lattices that contained the control or GW4064-treated HSCs were prepared as described in *Materials & Methods*. Hydrated collagen lattices were photographed 6 hours after addition of ET-1 (10 nM). Data are mean ±SE of 6 experiments. *P<.001 vs. control cells.

**Figure 4:**
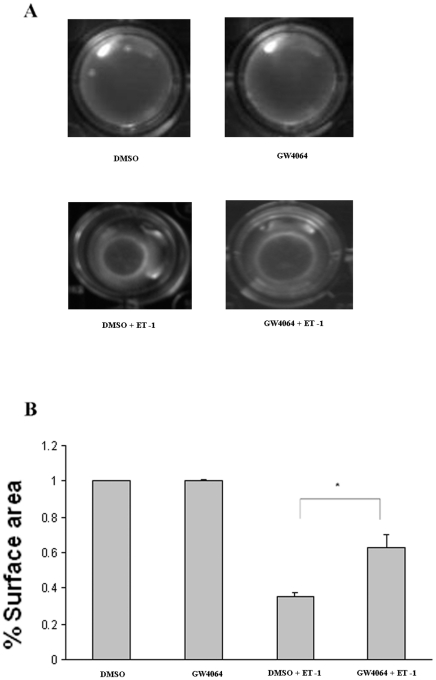
GW4064 treatment inhibited ET-1-induced contraction in fully activated HSCs. Rat HSCs were isolated and then cultured for 7 days to allow full activation. Collagen gel lattices that contained fully activated HSCs were then prepared. They were treated with DMSO or 1 µmol/L GW4064 for 18 hours. Hydrated collagen lattices were photographed 6 hours after addition of ET-1 (10 nM). Data are mean ±SE of 6 experiments. *P<.001 vs. control cells.

Following the demonstration that GW4064 treatment inhibited the transdifferentiation of HSCs and the associated ET-1 upregulation, we then investigated how the GW4064-treated HSCs responded to the ET-1-induced contraction and compared to that of untreated HSCs. HSC contraction was determined by collagen gel contraction assay. Fig. 3 shows that GW4064-treated HSCs were less active than DMSO-treated cells in response to ET-1-induced contraction. This is likely due to the fact that GW4064-treated cells are less transdifferentiated and are equipped with less active contractile machinery/mechanism. To study if GW4064 has a similar inhibitory effect on fully activated HSCs, freshly isolated HSCs were cultured for 7–10 days to allow full activation. The cells were then treated with GW4064 or DMSO for 18 h prior to ET-1-induced contraction. As shown in Fig. 4, transient treatment with GW4064 also partially blocked the ET-1-mediated contraction in fully activated HSCs. A similar result was obtained with LX-1, a human HSC cell line (data not shown).

### GW4064 treatment inhibited ET-1-mediated activation of RhoA/Rho-kinase signaling

The above studies clearly showed that GW4064 exerted a negative regulation of HSC contraction. To understand the underlying mechanism, we investigated the effect of GW4064 treatment on RhoA/Rho-kinase signaling as this pathway plays an important role in the contraction of activated HSCs. Initial study with real time RT-PCR showed that there was no difference in the mRNA expression levels of RhoA and its effector, Rho-kinase between GW4064- and DMSO-treated cells (data not shown), ruling out the possibility that FXR ligands affect HSC contraction via regulating the expression levels of the components in the RhoA/Rho-kinase signaling.

**Figure 5:**
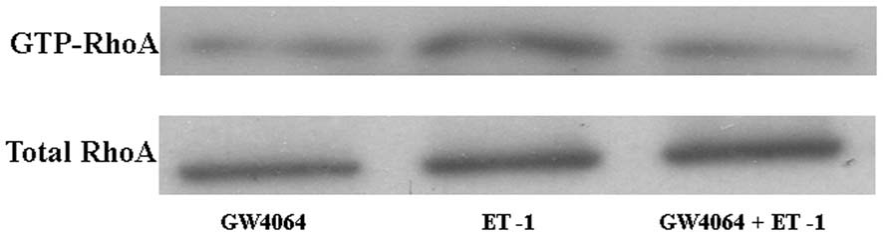
GW4064 treatment inhibited ET-1-induced RhoA activation in activated HSCs. Activated HSCs were treated with DMSO or 1 µmol/L GW4064 for 18 hours. ET-1 (10 nM) was then added to the medium, and the cells were harvested 10 min later. RhoA-GTP pull-down assay was performed using RhoA-GTP assay kit. RhoA-GTP pull-down and total RhoA fractions were probed by Western blot with anti-RhoA antibody. Blots are representative of experiments performed three times with similar results.

We then studied whether ET-1-induced activation of RhoA was altered in HSCs following treatment with GW4064. RhoA activities were determined by pull-down of GTP-RhoA. As shown in Fig. 5, stimulation of activated rat HSCs with ET-1 induced a significant increase in GTP-RhoA levels. Such activation of RhoA was significantly inhibited by pretreatment of the cells with GW4064 (Fig. 5).

**Figure 6:**
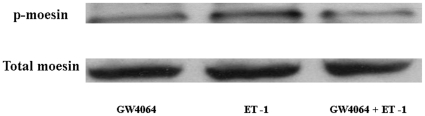
GW4064 treatment inhibited ET-1-induced Rho-kinase activity in activated HSCs. Activated HSCs were treated with DMSO or 1 µmol/L GW4064 for 18 hours. ET-1 (10 nM) was then added to the medium, and the cells were harvested 10 min later. Phosphorylation of meosin was detected by Western using appropriate phospho-specific antibody. An immunoblot of total meosin present in the cell extracts is shown as a loading control (lower panel). A representative immunoblot from three independent experiments is presented.

To test if the reduced ET-1-stimulated RhoA activation in GW4064-treated HSCs affects Rho-kinase activity, we studied the phosphorylation of the Rho-kinase substrate moesin at Thr-558 with a site- and phosphor-specific antibody. As shown in Fig. 6, ET-1 stimulation led to increased amounts of phosphorylated moesin in activated rat HSCs. Again, the ET-1-induced phosphorylation of moesin was significantly inhibited when the cells were pretreated with GW4064.

**Figure 7:**
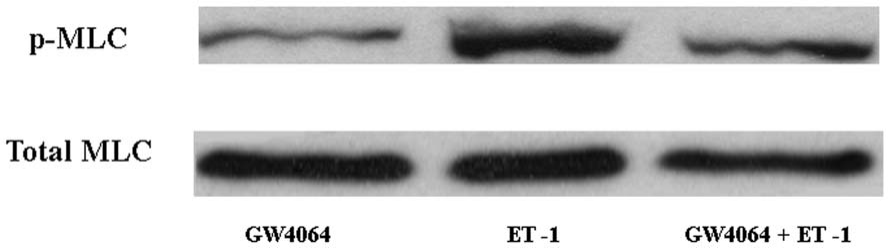
GW4064 treatment inhibited ET-1-induced phosphorylation of MLC in activated HSCs. Activated HSCs were treated with DMSO or 1 µmol/L GW4064 for 18 hours. ET-1 (10 nM) was then added to the medium, and the cells were harvested 10 min later. Phosphorylation of MLC was detected by Western using appropriate phospho-specific antibody. An immunoblot of total MLC present in the cell extracts is shown as a loading control (lower panel). A representative immunoblot from three independent experiments is presented.

One of the major consequences of activation of RhoA/Rho kinase pathway is the phosphorylation of myosin light chains (MLC), which is critically involved in the contraction of HSCs. To further elucidate the mechanism by which GW4064 inhibits HSC contraction, we studied if GW4064 affects the ET-1-induced phosphorylation of MLC. As shown in Fig. 7, exposure of the activated HSCs to ET-1 resulted in increased amounts of phosphorylated MLC, which was significantly inhibited by GW4064 pretreatment. The above results strongly suggest that GW4064 negatively regulates the contractile response of HSCs to ET-1 via inhibiting the RhoA/Rho kinase/MLC pathway.

## Discussion

We have confirmed and expanded previous reports that activation of FXR in HSCs inhibits their transdifferentiation. We have also shown for the first time that FXR ligands exert inhibitory effect on their contractile response to ET-1, a potent vasoconstrictor.

It has been well established that activation of HSCs plays a key role in the development of cirrhosis [1]. Activation of HSCs is associated with altered and increased production of ECM that contributes significantly to the fibrotic changes in the chronic liver injury. Increasing evidence also suggests that activated HSCs gains contractile function, which contributes to the intrahepatic hemodynamic changes in cirrhosis [19]. HSCs reside in the perisinusoidal space and extend elongate protrusions that run along and encircle one or more sinusoids. Activated stellate cells express α-smooth muscle actin, a marker of nonmuscle cell contractility, in patients with different types of chronic liver injury. A number of studies have demonstrated HSC contractile response following stimulation by many different vasoactive mediators including ET-1, arginine vasopressin, angiotensin II, thrombin, eicosanoids, and α1-adrenergic agonists [4], [5], [6], [20], [21]. The best-studied and most potent agonist for stellate cell contraction is ET-1. ET-1 was originally identified as a potent vasoconstrictor produced mainly by endothelial cells. During liver injury activated stellate cells become a major source of ET-1. Stellate cells also express endothelin receptors and ET-1 has a prominent contractile effect on stellate cells and myofibroblasts, which may contribute to portal hypertension in the cirrhotic liver. In addition, ET-1 promotes the proliferation of early-cultured stellate cells, whereas it inhibits fully activated ones [22]. Thus, ET-1 exhibits an autocrine effect on HSC and is involved in both HSC activation and their contractile response.

A number of nuclear receptors have been reported to show inhibitory effects on HSC activation. These include retinoid X receptor, PPARs, pregnane-X-receptor, and FXR [7], [8], [9], [23]. Treatment with the respective ligands has been shown to inhibit the activation of the stellate cells and decrease the fibrotic changes in animal models of liver injury. However little information is available on the roles of nuclear receptors in the regulation of stellate cell contraction.

As an initial step to address this question we studied the effect of GW4064, a synthetic FXR ligand, on the expression of ET-1 and its corresponding receptors on stellate cells. As shown in Fig. 2, there was a significant increase in the mRNA expression level of ET-1 during the process of stellate cell activation. This upregulation in ET-1 expression was significantly inhibited by GW4064 treatment. We have previously shown that activation of FXR inhibited both basal and LPS-stimulated ET-1 production in endothelial cells. Functional promoter assays including electrophoretic mobility shift assay (EMSA) and chromatin immunoprecipitation (ChIP) assay suggest that FXR inhibits ET-1 expression via interference with NF-κB/AP-1 signaling [24]. It is likely that FXR regulates ET-1 expression in stellate cells via a similar mechanism.

Despite the inhibitory effect on ET-1 expression, GW4064 showed no effect on the expression of either ETA or ETB receptor in stellate cells. A study by Chi and colleagues showed that retinoic acid exerted an inhibitory effect on ETB expression in stellate cells but showed no effect on either ET-1 or ETA expression [18]. Clearly, different nuclear receptors regulate stellate cell activation via different mechanisms.

The freshly isolated stellate cells that were continuously treated with GW4064 for 7 days showed reduced contractile response to ET-1. This is unlikely due to changes in the expression levels of ET-1 receptors as shown in our RT-PCR studies. It is likely due to the fact that GW4064-treated cells are less activated and are equipped with less active contractile mechanism. It has been shown that α-SMA is involved in the stellate cell contraction [25]. GW4064-treated cells have significantly reduced levels of α-SMA expression compared to fully activated stellate cells (Figure 1), which could account for, at least partially, the reduced contractile response to ET-1.

After demonstrating a reduced contractile response in GW4064-treated, partially activated stellate cells we further observed a similar inhibitory effect of GW4064 in fully activated stellate cells. To understand the underlying mechanism we focused on examining the effect of GW4064 on RhoA/Rho kinase signaling as this pathway plays a key role in stellate cell contraction [26]. Our preliminary study with quantitative RT-PCR showed no effect of GW4064 on the mRNA expression levels of either ETA or ETB. Thus it is unlikely that GW4064 inhibits stellate cell contractile response to ET-1 via modulating the expression of ET receptors at transcriptional level.

We then examined if GW6064 affects RhoA activation. Western analysis clearly showed that the ET-1-mediated activation of RhoA, measured as pull down of active GTP-RhoA, was significantly inhibited by GW4064 treatment. So far, the mechanism responsible for this inhibition is unclear. Preliminary studies showed that there were no significant changes in the mRNA expression levels of the receptors and several GTPase-activating proteins (p190 A-RhoGAP and p190 B-RhoGAP) and guanine nucleotide exchange factors (p115RhoGEF, PDZ-RhoGEF, and LARG) following treatment with GW4064 (data not shown). It remains to be determined if GW4064 treatment can induce any changes in the expression of these molecules at protein level. Nonetheless, the impaired activation of RhoA clearly resulted in a reduced activity of Rho-kinase, as shown by reduced phosphorylation of moesin, a member of the family of ezrin-radixin-moesin (ERM) proteins, which is phosphorylated at Thr-558 by Rho-kinase.

The inhibition of RhoA/Rho kinase signaling is clearly implicated in the GW4064-mediated inhibition of stellate cell contraction as the phosphorylation of the downstream MLC is significantly decreased. Many studies have shown that, similar to smooth muscle cells, contraction of stellate cells is powered by myosin II through its action on the actin cytoskeleton, a process that is activated by phosphorylation of its myosin regulatory light chain [27]. It's likely that inhibition of ET-1-induced MLC phosphorylation by GW4064 plays an important role in its inhibitory effect on stellate cell contraction.

In summary, in addition to its known anti-fibrotic effect, our study suggests a novel function of FXR in regulating intrahepatic vascular resistance through its inhibitory effect on stellate cell contraction. Although more studies are required to better understand the underlying mechanism, inhibition of RhoA/Rho kinase is likely to play a role in the FXR-mediated negative regulation of stellate cell contraction.

## Materials and Methods

### Ethics Statement

All experimental manipulations and protocols in this study were approved by the University of Pittsburgh Institutional Animal Care and Use Committee. The animals were housed and maintained in accordance with standards established in the Animal Welfare Act and the Guide for the Care and Use of Laboratory Animals (permit number: 0706848).

### Animals and HSC isolation

Retired male Sprague-Dawley rats were used for primary HSC isolation. HSCs were isolated via *in situ* proteinase/collagenase perfusion followed by density gradient centrifugation as described [28]. Primary cells were used at 5–7 days and were more than 95% pure. Cells were grown on standard tissue culture plastic dishes in DMEM with 10% fetal bovine serum and antibiotics.

### Quantitative real-time RT-PCR assay of gene expression

Total RNA was extracted from cells with TRIzol reagent (Invitrogen) and the first-strand cDNA was synthesized by use of SuperScript III reverse transcriptase (Invitrogen). Real-time PCR analysis of rat genes was performed by use of SYBR Green-based assays with the ABI 7300 Real-Time PCR System (Applied Biosystems, Foster City, CA) [29]. Each amplification mixture (25 µl) contains 25 ng of cDNA, 1.25 µl of primers and FAM-labeled florigenic probe, and 12.5 µl of Universal PCR Master mix. Amplification was performed under the following conditions: 50°C for 2 min, 95°C for 10 min, and 40 cycles of 95°C for 15 s, and 60°C for 1 min. Transcript abundance, normalized to β-glucuronidase expression, was expressed as fold increase over a calibrated sample.

### Immunostaining of cultured rat HSCs

Immunodetection of actin2 was performed following our published protocol [24]. Briefly, the cells that were grown on glass cover slips were washed three times with phosphate-buffered saline (PBS) and fixed with 4% (w/v) paraformaldehyde in PBS for 15 minutes at room temperature. After fixation, cells were rinsed with PBS and then permeabilized with 0.1% (v/v) Triton X-100, 0.1% (w/v) sodium citrate for 2 minutes on ice. Unspecific binding sites were blocked with 10% (v/v) goat serum in PBS, 0.1% (w/v) bovine serum albumin (BSA). Cells were then incubated for 2 hours at room temperature with rabbit anti-rat actin2 (Santa Cruz Biotechnology, Santa Cruz, CA) for 1 h, followed by labeling with Alexa 488-conjugated goat anti-rabbit IgG (Molecular Probes, Eugene, OR) for 1 h. Cells were observed under a confocal microscope.

### Western blot analysis for moesin and MLC protein expression

Protein extraction and Western blot analysis were performed as described previously [30]. Goat anti-moesin and anti-p-moesin antibodies were purchased from Santa Cruz Biotechnology, Inc. (Santa Cruz, CA). Rabbit anti-MLC and p-MLC antibodies were purchased from Cell Signaling Technology, Inc. (Danvers, MA). Horseradish peroxidase-labeled donkey anti-goat, goat anti-rabbit and the electrochemiluminescence kit were purchased from GE Healthcare (Piscataway, NJ).

### Pull-down assays for active RhoA

Cells were treated with and without ET-1 (10 nM) for 10 min for all experiments in which RhoA was assayed. To the diluted buffer (Upstate, Temecula, CA) in water, 10 µg/mL of aprotinin and 10 µg/mL leupeptin from Roche Molecular Biochemicals (Chicago, IL) were added. Cells were rinsed twice with PBS, and lysis buffer was added. Rho reagent slurry (Upstate) was added, samples were incubated, and beads were pelleted via brief centrifugation. Beads were resuspended in Laemmli-reducing buffer from Invitrogen (Carlsbad, CA) and boiled for 5 minutes. Supernatant and agarose pellets were mixed, and samples were loaded on sodium dodecyl sulfate-polyacrylamide gel electrophoresis gel (Invitrogen) and blotted to a nitrocellulose membrane. Anti-Rho antibody and mouse monoclonal IgG1 (Upstate) were applied for overnight, and appropriate secondary antibody was used for 1 hour. Chemoiluminescent from Pierce (Rockford, IL) was applied to the membrane for 5 minutes and developed on radiographic film.

### Gel contraction assay

Contractility of HSCs was evaluated using collagen gel lattices on 24-well culture plates as described [31]. Wells were filled with PBS containing 1% bovine serum albumin for 1 hour at 37°C, washed twice with PBS, and air-dried. Type1 rat-tail collagen (3.69 mg/mL; BD Bioscience, Bedford, MA) was adjusted according to the manufacturer's instructions. The final collagen concentration was 2 mg/mL. The collagen solution was poured and incubated for 1 hour at 37°C to allow gelation. HSCs were isolated and cultured as described. After 7 days of culture, cells were trypsinized, suspended (2.5×10^5^ cells/mL) in DMEM supplemented with 10% fetal bovine serum and antibiotics, and plated on the collagen gels (2 mL cell suspension/well). After incubation overnight to allow cell attachment, serum-free conditions were introduced for 4 hours and the cells were washed three times with serum-free DMEM. Gels were detached from the plates. HSCs were treated with GW4064 for 18 hour. ET-1 (10 nM) was then added to the culture media just before the lattices were detached. Buffer without ET-1 was used as a control. The gels were photographed every hour for 6 hours. Surface area of the collagen gels was measured using digital image analysis software. All experiments were performed as triplicate.

### Statistical analysis

All data are expressed as means ± S.E.M. unless otherwise stated. Comparisons between two groups were made with unpaired Student's *t* test. Comparisons between three or more groups were made with analysis of variance followed by Tukey-Kramer post hoc analysis. In all cases, *P*<0.05 was considered statistically significant.
